# Twice-weekly aripiprazole for treating children and adolescents with tic disorder, a randomized controlled clinical trial

**DOI:** 10.1186/s12991-016-0112-4

**Published:** 2016-08-30

**Authors:** Ahmad Ghanizadeh

**Affiliations:** 1 Research Center for Psychiatry and Behavioral Sciences, Shiraz University of Medical Sciences, School of Medicine, Shiraz, Iran; 2 Department of Psychiatry, Shiraz University of Medical Sciences, School of Medicine, Shiraz, Iran; 3 Department of Neuroscience, School of Advanced Medical Sciences and Technologies, Shiraz University of Medical Sciences, Shiraz, Iran; 4 Substance Abuse Research Center, Shiraz University of Medical Sciences, Shiraz, Iran; 5 Department of Psychiatry, Research Center for Psychiatry and Behavioral Sciences, Hafez Hospital, Shiraz, Iran

**Keywords:** Aripiprazole, Treating, Tic disorder, Clinical trial

## Abstract

**Objective:**

Treating tic disorder is challenging. No trial has ever examined whether twice weekly aripiprazole is effective for treating tic disorders.

**Methods:**

Participants of this 8-week randomized controlled parallel-group clinical trial were a clinical sample of 36 children and adolescents with tic disorder. Yale global tic severity scale was used to assess the outcome. Both groups received daily dosage of aripiprazole for the first 14 days. Then, one group received daily dose of aripiprazole while the other group received twice weekly dosage of aripiprazole for the next 46 days. The patients were assessed at baseline, week 2, 4, and 8.

**Results:**

Tic scores decreased in both group significantly 22.8 (18.5) versus 22.0 (11.6). Moreover, there was no between group difference. The final mean (SD) score of motor and vocal tics in the group treated with daily treatment was not significantly different from the twice weekly group (Cohen’s *d* = 0.36). The odds ratios for sedation and increased appetite were 3.05 and 3, respectively.

**Discussion:**

For the first time, current findings support that twice weekly aripiprazole efficacy was not different from that of daily treatment. The rate of drowsiness in the twice weekly treatment group was less than that of the daily treatment group.

This trial was registered at http://www.irct.ir. The registration number of this trial was: IRCT201312263930N32. http://www.irct.ir/searchresult.php?id=3930&number=32

## Background

The neurodevelopmental disorder of Tourette’s syndrome is usually chronic with both motor and vocal tics. Its rate in boys is higher than that in girls [1]. Not only the complexity of tic disorders but also their treatments and the effectiveness and adverse effects of the medications are major challenges for specialists [2, 3]. There is no medication to ameliorate all the symptoms of tic disorder in all patients. Behavior therapy reduces tic symptom severity in patients with tic disorders [4]. Habit reversal training is a type of behavior therapy. It markedly reduces tic severity [5]. Psycho-education is a major part of treatment to increase the tolerance of the patients. Pharmacotherapy does not cure tic symptoms but it may control or decrease some symptoms.

While there is no consensus about treating tic disorders with medications, pharmacotherapy usually includes dopamine antagonists such as typical and atypical antipsychotics (such as aripiprazole), and α-2-adrenoreceptor agonists (e.g., clonidine).

In addition to concerns about the efficacy, antipsychotic related adverse effects such as extrapyramidal symptoms, increased prolactin, sedation and increased weight are among the concerns for pharmacotherapy.

Aripiprazole has been introduced for treating tic disorders [2]. There is good evidence regarding its effectiveness and safety in tic disorder [6]. A 10-week multicenter, double-blind, randomized, placebo-controlled trial on 61 children and adolescents with the diagnosis of Tourette’s disorder showed that aripiprazole in comparison to placebo was effective and relatively safe in the short-term treatment [7]. Aripiprazole decreased 15 score from the score of YGTS scale [7]. Other uncontrolled and controlled studies also support these findings in children and adolescents [8–10].

Aripiprazole [3.22 (1.9) mg/day] is as effective as risperidone [0.6 (0.2) mg/day] for treating tic disorders [11]. Moreover, the safety of these two medications is comparable in children and adolescents with tic disorders [11].

The oral availability of aripiprazole is 87 % [12]. The plasma concentrations of aripiprazole and its active metabolite both reach a steady state by Day 14 after repeated oral administration [13]. Its mean elimination half-life ranges from 47 to 68 h [14]. Another study reported that its mean elimination half-life was about 75 h while the half-life of its active metabolite was 94 h [12].

Because of the long half-life of aripiprazole, it is administered daily although it might be administered twice-weekly. Adverse effects are major concern for treating tic disorders with medications. Many of these adverse effects are dose dependent. For example, sedation and increased appetite are common. Both of these adverse effects negatively impact educational and social functioning of students. Sometimes, these adverse effects might decrease treatment adherence. We could not find any trial examined whether twice-weekly administration of aripiprazole is effective and safe as much as its daily administration.

It is hypothesized that twice-weekly aripiprazole as much as daily aripiprazole is effective for treating tic disorders.

## Methods

Participants of this 8-week randomized controlled parallel-group clinical trial were a clinical sample of 46 children and adolescents with tic disorder aged less than 19 years old. The diagnoses of tic disorders were made using DSM-IV diagnostic criteria by a board certified child and adolescent psychiatrist. The severity of tic disorder was measured using a semi-structured clinical interview, the Yale global tic severity scale (YGTSS) [15].

Adverse effects were checked using a checklist based on the most common adverse effects of aripiprazole. Patient or parent-reported adverse effects were also recorded [11]. Concomitant medications were documented.

Patients were randomly allocated into one of the two groups. Random numbers were provided by a random number generator. Both groups received aripiprazole (starting dose of 1.25 mg/day and is titrated up to 7.5 mg/day during 1 week) [16]. Both groups received daily dosage of aripiprazole for the first 14 days. Then, one group received daily dose of aripiprazole while the other group received twice weekly dosage of aripiprazole for the next 46 days. Dosage could be adjusted according to side effects. Psychotropic drugs to control comorbid psychiatric symptoms were allowed during the trial. Assessments were occurred at pre-intervention, week 2, 4, and 8. Medication adherence was checked through asking the patients and the parents.

The exclusion criteria were current mood disorders, psychotic symptoms, severe uncontrolled general medical conditions or neurological problems and such as diabetes, epilepsy, Huntington’s chorea, reported cardiac problems, and clinically estimated mental retardation. Patients were drug free in the last 2 weeks prior entry into this trial. Otherwise, there was no significant dosage change in the last 2 weeks or during the trial.

Subjects were both males and females, aged 6–18 years old. YGTSS score more than 21 on YGTSS was required to enter this trial or tics had to be severe producing marked parent-or subject-reported distress or impairment [11]. This score is considered as a moderate severity [16].

Ethics Committee of Shiraz University of Medical Sciences approved this trial (no. 6631). The assent and written informed consent were provided by the children and their parents, respectively.

The total tic subscale of Yale global tic severity scale score was our main outcome measurement [15]. It consists of the total score of both vocal and motor tic severity ranging from 0 to 50. Total Yale global tic severity scale score consists of Total tic severity score plus the score of impairment related to the vocal and motor tics. Its score ranges from 0 to 100.

Similar to our previous trial [11], at least 35 % decline considered as a treatment response. However, complete response was defined as more than 50 % decline in tic symptoms as measured on the YGTSS.

A previous clinical trial showed that the daily administration of aripiprazole decreased total tic severity score from 16.5 (6.4) to 5.7 (6.2) during 2 months [11]. Another trial showed that aripiprazole declined 15 score from YGTSS [7]. Therefore, a total of 20 patients (10 patients in each group) is required to be entered in this two-treatment parallel-design trial based on the assumption of the true difference between treatments 9.0 units, standard deviation = 6, *P* = 0.05, and power = 85 %.

### Data analysis

SPSS for Windows was used to run the statistical analysis. Sample Kolmogorov–Smirnov Test was performed to test normal distribution (*P* = 0.97). Gender ratio and response rates were compared between the two groups using Fisher’s exact test or Chi-Square test, whenever it was applicable. *t* test was used to compare the mean of age and the decline of YGTSS between the two groups. Repeated measure analysis of variance (ANOVA) was performed to evaluate the overall efficacy rate. Cohen’s *d* was used to assess effect size. Relative risk ratio was used to compare the effect size the two treatments for complete remission. Intention-to-treat analysis (ITT) with last-observation-carried-forward (LOCF) for the patients who had been assessed for at least 2 sessions was performed to handle the missing data.

*P* value less than 0.05 was set as the statistically significant difference.

## Results

**Fig. 1:**
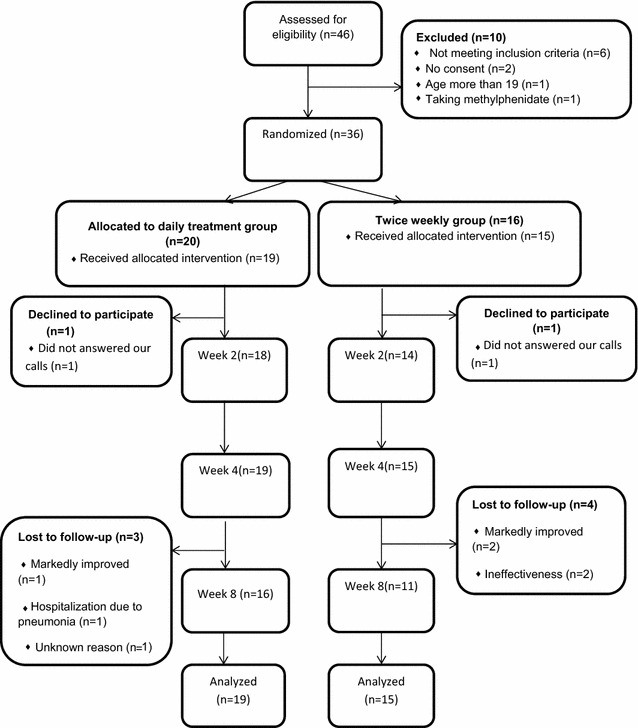
The CONSORT flow diagram of the patients in this trial

The characteristics of the patients are displayed in Table 1. Gender ratio and mean age were not different between the two groups. The rates of co-morbid psychiatry disorders were not different between the two groups (Table 1). The frequency of concurrent medications was not different between the two groups too (Table 1). Out of 46 patients were screened for entering this trial, ten patients were excluded due to inclusion or exclusion criteria. The reasons for the exclusions are presented in the CONSORT flow diagram (Fig. 1). One patient in the daily treatment group and one patient in the twice weekly group withdrew their consent for participation.

**Table 1:** Characteristics of the patients in the daily treatment group and twice weekly treatment group

	Daily treatment group	Twice weekly treatment group	Significance
Mean (SD) years of age	10.5 (3.1)	10.8 (2.0)	*t* = .3, d*f* = 34, *P* = 0.72
*Gender*			
Boys	16 (80 %)	14 (87.5 %)	*P* = 0.67
Girls	4 (20 %)	2 (12.5 %)	
*Comorbid psychiatric disorder*			
ADHD	6 (37.5 %)	5 (41.7 %)	*X*2 = 0.05, d*f* = 1, *P* = 0.8
Oppositional defiant disorder	7 (43.8 %)	7 (58.3 %)	*X*2 = 0.5, d*f* = 1, *P* = 0.4
Separation anxiety disorder	0	2 (16.7 %)	–
Obsessive compulsive disorder	1 (6.2 %)	0	–
*Con-current medications (n)*			
Carbamazepine	1	–	–
Ritalin (10 mg/day)	1	–	–
Imipramine (10 mg/day) + haloperidole (0.5 mg/day)	–	1	–
*Mean dosage of aripiprazole*			
First 2 weeks	3.5	3.8	–
Second 2 weeks	3.8	4.6	–
Second month	4.0	4.5	–
*Motor and vocal tics score*			
Baseline	33.6 (18.2)	31.3 (7.6)	*t* = .4, d*f* = 34, *P* = 0.6
Week 2	15.1 (10.8)	12.8 (9.1)	*t* = 0.6, d*f* = 32, *P* = 0.52
Week 4	11.0 (9.7)	12.8 (10.9)	*t* = 0.5, d*f* = 31, *P* = 0.6
Week 8	6.6 (5.9)	9.7 (11.2)	*t* = 1.03, d*f* = 32, *P* = 0.3
Mean (SD) decline of total Yale motor and vocal tic severity scale score during trial	22.8 (18.5)	22.0 (11.6)	*t* = 1.07, d*f* = 32, *P* = 0.2
Response rate with more than 35 % decline in total Yale motor and vocal tic severity scale score	19 (100.0 %)	11 (73.3 %)	*P* < 0.02
Response rate with more than 50 % decline in total Yale motor and vocal tic severity scale score	17 (89.5 %)	11 (73.3 %)	*P* = 0.2
Total Yale global tic severity scale score (motor tics + vocal tics + impairments)			
Baseline	89.6 (39.2)	93.8 (29.3)	*t* = 0.3, d*f* = 34, *P* = 0.72
Week 2	39.5 (33.33)	37.8 (31.3)	*t* = 0.1, d*f* = 32, *P* = 0.8
Week 4	27.1 (31.9)	39.2 (36.7)	*t* = 1.0, d*f* = 31, *P* = 0.31
Week 8	15.3 (17.2)	29.7 (37.1)	*t* = 1.4, d*f* = 32, *P* = 0.14
Mean (SD) decline of total Yale global tic severity scale score during trial (motor tics + vocal tics + impairments)	76 (37.9)	64.6 (44.2)	*t* = 0.8, d*f* = 32, *P* = 0.4
Response rate with more than 35 % decline in total Yale global tic severity scale score (motor tics + vocal tics + impairments)	19 (100.0 %)	11 (73.3 %)	*P* < 0.02
Response rate with more than 50 % decline in total Yale global tic severity scale score (motor tics + vocal tics + impairments)	17 (89.5 %)	11 (73.3 %)	*P* = 0.3

Four patients in the twice weekly group dropped out at week 4. The reasons for the dropout were: being ineffective (*n* = 2), and markedly improved (*n* = 2).

Three patients in the daily treatment group did not complete this trial. All of them dropped out at week 4. One of them dropped out because the patients markedly improved. The other one dropped out with an unknown reason. The reason for another patient was hospitalization due to pneumonia.

### Total Yale global tic severity scale score (motor tics + vocal tics + impairments)

The mean of Total Yale global tic severity scale score at baseline in the daily treatment group and twice weekly treatment group was 89.6 (39.2) and 93.8 (29.3), respectively (*t* = 0.3, d*f* = 34, *P* = 0.72) (Table 1).

**Fig. 2:**
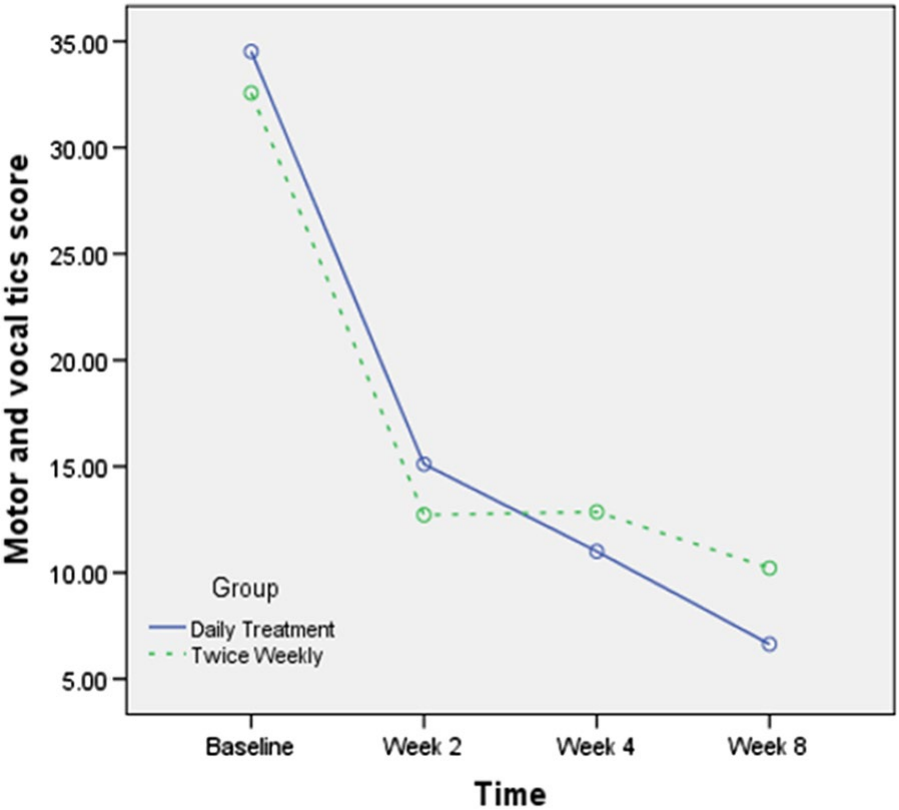
Changes of the sum up of motor and vocal tics score during this trial by the groups

The results of Repeated-measure ANOVA showed significant effect for time during this trial (Sphericity Assumed: F3, 93 = 56.8, *P* < 0.001) (Fig. 2). The interaction of time × treatment was not statistically significant (Sphericity Assumed: F3, 93 = 0.74, *P* = 0.4). There was no between group difference (F1, 31 = 0.85, *P* = 0.36). At the end of this trial, the mean decline of TYGTSS in the daily treatment group and twice weekly treatment group was not different between the two groups [76 (37.9) versus 64.6 (44.2), respectively, *t* = 0.8, d*f* = 32, *P* = 0.4] (Table 1).

By week 8, the rate of partial response was 100.0 % in the daily treatment group. The rate in the twice weekly treatment group was 73.3 % (*P* < 0.02). However, the rate of complete response (more than 50 % decline in TYGTSS was not different between the two groups. The rate in the daily treatment group was 89.5 % while it was 73.3 % in the twice weekly group. These rates were not different between the two groups (*P* = 0.3). The response rates yield a relative risk ratio of 1.22.

### Total Yale tic severity scale score (motor tics + vocal tics)

The mean score of TYTSS was not different between the two groups at baseline (Table 1). TYTSS decreased 27.8 (18.5) and 22.0 (11.6) in the daily treatment group and twice weekly treatment group, respectively.

Repeated-measure ANOVA displayed that there was a significant effect for time during this trial (Sphericity Assumed: F3, 93 = 48.7, *P* < 0.001). Moreover, the interaction of time × treatment was not statistically significant (Sphericity Assumed: F3, 93 = 0.43, *P* = 0.43) (Table 1). In addition, there was no significant difference between the two groups (F1, 31 = 0.01, *P* = 0.92).

By week 8, while the rate of partial response was different between the two groups, the rate of complete response (more than 50 % decline in TYTSS) was not different between the two groups (*P* = 0.3).

The final mean (SD) score in the group treated with daily treatment was not significantly different from the twice weekly group [6.6 (5.9) versus 9.7 (1.2); *P* = 0.3], a difference of −3.1 and Cohen’s *d* = 0.36 [6.6–9.7 = −3.1; 3.1/8.5 (pooled SD) = 8.5].

### Vocal tics

**Fig. 3:**
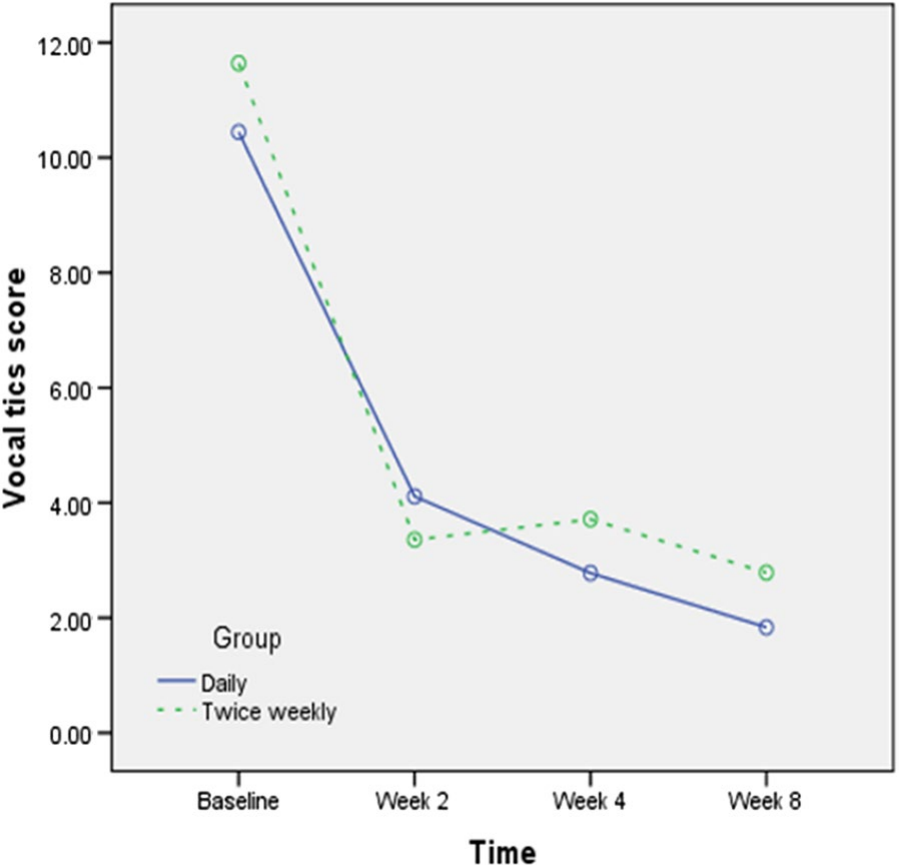
Changes of the vocal tics score during this trial by the groups

There was no between group difference regarding the effect on the vocal tice (F1, 30 = 0.11, *P* = 0.7) (Fig. 3). The interaction of time × treatment was not statistically significant (F3, 90 = 0.32, *P* = 0.68).

### Motor tics

**Fig. 4:**
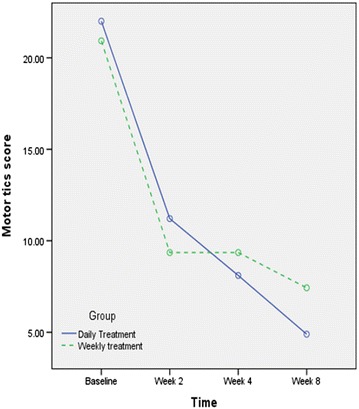
Changes of the motor tics score during this trial by the groups

As it is displayed in the Fig. 4, the effect of groups on the motor tics score was not different (F1, 31 = 0.02, *P* = 0.8). The interaction of time × treatment was statistically non-significant (F3, 33 = 1.43, *P* = 0.24).

### Adverse effect

The most common adverse effect was drowsiness. The rate of drowsiness in the daily treatment group and twice weekly group was 52.6 and 26.7 %, respectively (Table 2). The odds ratio for sedation was 3.05 (95 % Confidence Interval: from 0.71 to 13.10). The odds ratio for increase appetite was 3 (95 % Confidence Interval: from 0.50 to 17.70). The other most common adverse effects in the daily treatment group were increased appetite (31.6 %), fatigues (10.5 %), irritability (10.5 %), and headache (10.5 %). The most common adverse effects in the twice weekly group were drowsiness (26.7 %), increased appetite (10.3), fatigue (10.3), and nausea (10.3).

**Table 2:** The rate of adverse effects by the groups

Adverse effect	Daily treatment group		Twice weekly group		Significance
	*n*	%	*n*	%	
Drowsiness	10	52.6	4	26.7	*X*2 = 0.12, d*f* = 1, *P* = 0.1
Increased appetite	6	31.6	2	10.3	*X*2 = 0.2, d*f* = 1, *P* = 0.2
Fatigue	2	10.5 %	2	10.3	–
Irritability	2	10.5 %	0	–	–
Headache	2	10.5 %	2	10.3	–
Nausea	1	5.3	2	10.3	–
Slowness	1	5.3	0	–	–
Abdominal pain	1	5.3	1	6.7	–
Dizziness	0	–	1	6.7	–
Drooling	1	5.3	0	–	–
Decreased appetite	0	–	0	–	–
Tremor	0	–	0	–	–
Itches	0	–	0	–	–
Convulsion	0	–	0	–	–
Akathisia	0	–	0	–	–
Dyskinesia	0	–	0	–	–
Diurnal urinary incontinency	0	–	0	–	–
Walking problem	0	–	0	–	–
Diarrhea	0	–	0	–	–
Vomiting	0	–	0	–	–
Insomnia	0	–	0	–	–
Swallowing difficulty	0	–	0	–	–
Blurred vision	0	–	0	–	–

## Discussion

Current results of this randomized, open label, control clinical trial suggest that twice weekly administration of aripiprazole is as effective as daily administration of aripiprazole in the reduction of tics in children and adolescents with Tourette’s disorder. Both treatment protocols significantly decreased the tics score. In addition, the rate of complete response was not different between the two groups. However, one group received aripiprazole every day while the other group received it twice weekly (every Saturdays and Tuesdays). The relative risk ratio of 1.22 shows that the probability of response to daily treatment is not markedly higher than that of the twice-weekly treatment.

These results support our hypothesis that twice weekly administration of aripiprazole is effective for treating Tourette’s disorder. This is in similar line with long half-life of aripiprazole and its metabolite. For the first time, current findings support that twice weekly aripiprazole efficacy was equivalent to that of daily treatment.

Patients in both groups tolerated the medication very well. The rate of adverse effects was similar between the two groups. The adverse effects were not severe and they were manageable well. No one dropped out due to adverse effects. The most common adverse effect was drowsiness. The rate of drowsiness in the twice weekly treatment group was half of the daily treatment group. The odds ratio of 3 for drowsiness and increased appetite indicates that the patients in the daily treatment group suffer from sedation and increased appetite more than three times from the control group. Current literature support that aripiprazole is commonly well tolerated [17] and it is considerably safe in children and adolescent [18]. Its adverse effects are mild to moderate and transient [17]. If further well controlled double blind controlled clinical trial support current finding, twice weekly administrating of aripiprazole is preferred in compare to daily administrating.

There are some limitations needed to be taken into account. This short term, small sample size, and open label trial was from a specialty clinic. It may reduce the generalizability of the results. In addition, the rater and the patients were not blinded to treatment group. Further trials should examine blood data including verifying the patients’ reports and indicating a profile how the drugs serum’s level develop and the degree of fluctuation despite the long half-life to a certain degree.

Despite these limitations, this is the first randomized controlled clinical trial examining the effectiveness and safety of twice weekly treatment of Tourette’s disorder with aripiprazole.

Current results encourage performing further large sample size, double blind randomized control clinical trials.

## Conclusion

According to the current results, the efficacy of daily treatment with aripiprazole was not different from the twice weekly treatment for treating Tourette’s disorder in children and adolescents.
